# LPRP: A Gene–Gene Interaction Network Construction Algorithm and Its Application in Breast Cancer Data Analysis

**DOI:** 10.1007/s12539-016-0185-4

**Published:** 2016-09-17

**Authors:** Lingtao Su, Xiangyu Meng, Qingshan Ma, Tian Bai, Guixia Liu

**Affiliations:** 1 College of Computer Science and Technology, Jilin University, Changchun, 130012 China; 2 Key Laboratory of Symbolic Computation and Knowledge Engineering of Ministry of Education, Jilin University, Changchun, 130012 China; 3 The First Clinical Hospital of Jilin University, Changchun, 130021 China

**Keywords:** Gene–gene interaction, Network construction, Breast cancer, TCGA dataset

## Abstract

The importance of the construction of gene–gene interaction (GGI) network to better understand breast cancer has previously been highlighted. In this study, we propose a novel GGI network construction method called linear and probabilistic relations prediction (LPRP) and used it for gaining system level insight into breast cancer mechanisms. We construct separate genome-wide GGI networks for tumor and normal breast samples, respectively, by applying LPRP on their gene expression datasets profiled by The Cancer Genome Atlas. According to our analysis, a large loss of gene interactions in the tumor GGI network was observed (7436; 88.7 % reduction), which also contained fewer functional genes (4757; 32 % reduction) than the normal network. Tumor GGI network was characterized by a bigger network diameter and a longer characteristic path length but a smaller clustering coefficient and much sparse network connections. In addition, many known cancer pathways, especially immune response pathways, are enriched by genes in the tumor GGI network. Furthermore, potential cancer genes are filtered in this study, which may act as drugs targeting genes. These findings will allow for a better understanding of breast cancer mechanisms.

**Electronic supplementary material:**

The online version of this article (doi:10.1007/s12539-016-0185-4) contains supplementary material, which is available to authorized users.

## Introduction

Breast cancer and many other malignancies result from stepwise genetic alterations of cells [1, 2]. Over the last decade, although the knowledge of specific genes and various biological pathways of breast cancer has been revealed, the understanding of breast cancer biology remains limited [3, 4]. In fact, single genes or protein alterations are not sufficient to induce cancer, but their interactions with other genes or their surroundings play key roles [5–7]. Performing network analysis using large-scale gene expression datasets is an effective way to uncover new biological knowledge. Network analysis has revolutionized our understanding of biological processes and made significant contributions to the discovery of disease biomarkers. Hence, to better understand cancer pathogenesis, research from network perspective is urgently needed [8–13].

Detecting pairwise interactions among genes plays basic roles in the construction of GGI network. Many GGI prediction methods have been proposed, including experimental methods such as affinity purification [14] and yeast two-hybrid assays [15], but such methods are generally in low efficiency and high cost. Recently, calculation-based gene correlation prediction methods incorporating gene expression datasets have been preferred [16, 17], such as Pearson’s, Spearman’s and Kendall’s correlations, distance correlation, Hoeffding’s D measure, Heller–Heller–Gorfine measure, mutual information (MI) [18] and maximal information coefficient (MIC) [19]. Pearson’s correlation is the most commonly used method for detecting linear relationships among genes. For nonlinear or non-functional relationship, rank correlation-based methods or information theory-based measures are more applicable. MIC is based on the idea that if a relationship exists between two random variables, then a grid can be drawn on the scatter plot of the two variables [20]. However, gene expression data contain various types of relationships, many of the methods only capture one type of interaction (promotion or suppression). In this study, we proposed a new GGI network construction approach termed linear and probabilistic relations prediction (LPRP). LPRP constructs GGI network in three steps: raw network construction, expansion and revision. During each step, only high-confidence gene interactions are considered, and a backbone network is utilized. The complete human protein interaction network from Pathway Commons [21] was used as the backbone network topological structure, and only interactions in the backbone network are kept to construct the raw GGI network. Such methodological approach has been proven fruitful in a variety of tumor genetic research fields [10, 22–27]. LPRP detects both linear and probabilistic relations among genes. In [28], the authors used a similar method but did not consider reverse regulation. In addition, we used a totally different gene interaction measure strategy.

In this study, we validate the effectiveness of LPRP using both simulated and real gene expression and applied LPRP on breast cancer data analysis. We construct separate genome-wide GGI networks for tumor and normal breast samples by applying LPRP on their gene expression datasets profiled by The Cancer Genome Atlas (TCGA). The identification of global gene interaction perturbations that actively participate in the initiation and maintenance of the tumor state is a major challenge in cancer biology [29]. Over the last decade, specific cancer genetic alterations have been well described and annotated [30], but network-level research has rarely been conducted. In this study, we performed a multi-level study (firstly, we compared the difference between normal and tumor GGI network from the gene-level, i.e., compare the difference between the gene interactions. Secondly, we compared the modularity difference between the constructed tumor and normal GGI network, i.e., cluster-level. Finally, we compared the network topology difference, i.e., network-level comparison). It is known that functionally related genes tend to cluster together in the biological network [31, 32]. Many network clustering algorithms [33–35] are available in this area. In this study, MINE [35] was used for cluster detection, as in many previous studies [36], which can be easily done using Cytoscape [37]. KEGG pathway enrichment analysis was performed using the SIGORA R package under the default parameter settings [38]. Furthermore, node degrees of many known tumor genes were compared, and by mapping known breast cancer genes to the tumor GGI network, some potential cancer genes were filtered, which may act as drugs targeting genes. Our findings allow for a better understanding of breast cancer mechanisms and may have potential implications for identifying novel drug targets.

## Methods and Materials

### Materials

UNC IlluminaHiSeq_RNASeqV2 level 3 (Refer to https://tcga-data.nci.nih.gov/tcgafiles/ftp_auth/distro_ftpusers/anonymous/tumor/read/cgcc/unc.edu/illuminahiseq_rnaseqv2/rnaseqv2/unc.edu_READ.IlluminaHiSeq_RNASeqV2.mage-tab.1.6.0/DESCRIPTION.txt for details of the RNASeqV2 pipeline and the algorithms) gene expression datasets of 20,502 genes, including 120 breast cancer samples and 106 normal samples, were downloaded from the TCGA project webpage. Raw count values were normalized using the TCGA-Assembler [40]. To further reduce the error, genes with values of 0 across all samples were deleted, leaving only 16,441 and 16,999 genes in the normal and tumor expression matrixes, respectively. The complete human protein–protein interaction network was downloaded from Pathway Commons (http://www.pathwaycommons.org/), which was generated by bringing together protein interactions from the following sources: the Human Protein Reference Database (HPRD) [41], the National Cancer Institute Nature Pathway Interaction Database (NCI-PID) [42], the Interactome (Intact) [43] and the Molecular Interaction Database (MINT) [44]. We focused on non-redundant interactions, only including proteins with an Entrez gene ID annotation, and isolated nodes or edges were deleted. As a result, we obtained a connected network with 15,589 nodes (unique Entrez IDs) and 1896,352 documented interactions. Hereafter, we refer to this network as the “KP”. For comparison, two random datasets were generated, and 237 known breast cancer-related genes were downloaded from SNP4Disease (http://snp4disease.mpi-bn.mpg.de/result.php). After deleting non-expressed genes, only 166 genes left. The gene expression dataset and its benchmark networks of the Dream5 challenge4 network inference challenge [45], downloaded from (http://wiki.c2b2.columbia.edu/dream/index.php?title=D5c4), were also used for comparison. To do systematic evaluation, two datasets are used. One is a real gene expression dataset contained in R package minet [46]. The other is a simulated dataset simulated by SynTReN (synthetic transcriptional regulatory network) [47] which has 100 genes and 100 samples.

### Methods

LPRP takes the discretized matrix ***D*** (constructed later) and ***KP*** as inputs and outputs the corresponding GGI network. LPRP works in the following steps: First, the gene expression matrix is discretized; second, gene interactions are detected and statistically validated; and third, the GGI network is constructed.

#### Discretization of Gene Expression Matrix

We denote the gene expression data matrix as ***M***. For each row of ***M***, its average and standard deviation are calculated as in [28]. We denote the average and deviation of the ***i****th* row as ***avg***_***i***_ and ***sd***_***i***_, respectively. ***D*** is defined by Eq. (1):

1 $$D_{ij} = \left\{ \begin{array}{l} - 1{\text{ if }}M_{ij} < {\text{avg}}_{i} - \gamma \times {\text{sd}}_{i} \hfill \\ 0{\text{ if avg}}_{i} - \gamma \times sd_{i} \le {\text{if }}M_{ij} \le {\text{avg}}_{i} + \gamma \times {\text{sd}}_{i} \hfill \\ 1{\text{ if }}M_{ij} > {\text{avg}}_{i} + \gamma \times {\text{sd}}_{i} \hfill \\ \end{array} \right.$$

where $\gamma$ is the threshold value between 0 and 1.

**Fig. 1:**
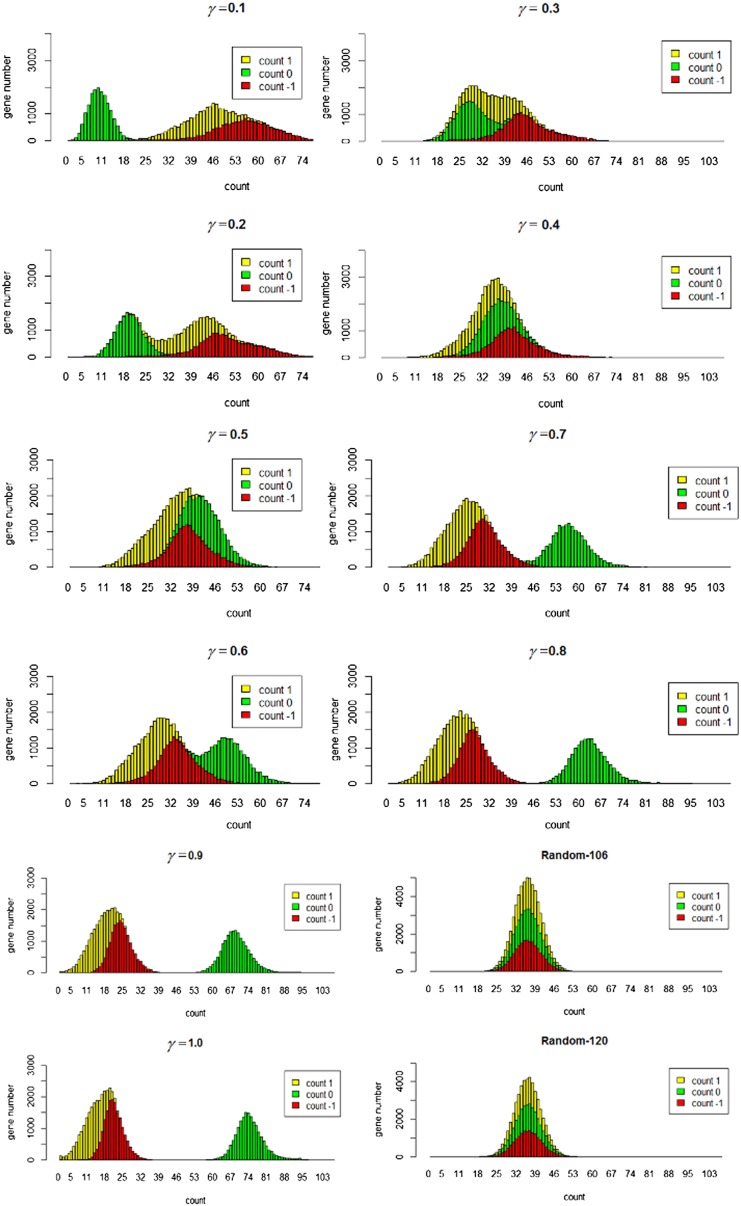
Frequency distribution of −1, 0, 1 s in the discretized matrix **D** and in the two random matrices

We vary $\gamma$ from 0 to 1 in steps of 0.1. For each case, the frequency distribution of the genes with respect to the counts of 1, 0, −1 s in their profiles is shown in Fig. 1. As shown in Fig. 1, when $\gamma$ takes a value between 0.4 and 0.5, the distribution that is most similar to the distribution of the randomly generated discretized matrix and also has similar distribution with those generated from currently commonly used methods in “sdnet” R package (randomly generated of 0, 1 and −1) shown in Fig. 1. Hence, in this work, we used $\gamma$ = 0.45 for the discretization of the gene expression matrix.

#### Gene Interaction Detection

Based on the discretized gene expression matrix ***D***, for each pair of genes in ***D*** under the same sample, only nine possible value combinations exist. We represent one gene as ***g***_***i***_ and another gene as ***g***_***j***_; such combinations are shown in Eq. (2):

2 $$\begin{aligned} C(g_{i} ,g_{j} ) = \left\{ \begin{aligned} ( - 1, - 1){\text{ where }}g_{i} = - 1{\text{ and }}g_{j} = - 1 \hfill \\ ( - 1, \, 0){\text{ where }}g_{i} = - 1{\text{ and }}g_{j} = 0 \hfill \\ ( - 1, \, 1){\text{ where }}g_{i} = - 1{\text{ and }}g_{j} = 1 \hfill \\ ( { }0, - 1){\text{ where }}g_{i} = 0{\text{ and }}g_{j} = - 1 \hfill \\ ( { }0, \, 0){\text{ where }}g_{i} = 0{\text{ and }}g_{j} = 0 \hfill \\ ( { }0, \, 1){\text{ where }}g_{i} = 0{\text{ and }}g_{j} = 1 \hfill \\ ( { }1, - 1){\text{ where }}g_{i} = 1{\text{ and }}g_{j} = - 1 \hfill \\ ( { }1, \, 0){\text{ where }}g_{i} = 1{\text{ and }}g_{j} = 0 \hfill \\ ( { }1, \, 1){\text{ where }}g_{i} = 1{\text{ and }}g_{j} = 1 \hfill \\ \end{aligned} \right. \hfill \\ \hfill \\ \end{aligned}$$

For each form of combination in Eq. (2), its probability value across all samples is calculated from Eq. (3):

3 $$P(v_{i} ,v_{j} ) = \frac{{\sum\limits_{h = 1}^{N} {D_{{g_{i,h} }} = v_{i} \wedge D_{{g_{j,h} }} = v_{j} } }}{N}$$

where ***N*** is the sample number in matrix ***D***, ***g***_***i***_ and ***g***_***j***_ are the two genes as in Eq. (2), ***v***_***i***_ and ***v***_***j***_ take values from −1, 0, 1.

For simplicity, only three forms of interactions between ***g***_***i***_ and ***g***_***j***_ are considered. That is, ***g***_***i***_ and ***g***_***j***_ are forward-regulated, reverse-regulated or have no interaction. Furthermore, we hypothesize that if ***g***_***i***_ and ***g***_***j***_ have a forward-regulated relationship then their reverse-regulated power is small or has no reverse regulation relationship between them and vice versa. Furthermore, considering the perspective of entire network, only one form of regulation dominates between the two genes, even though the other form of regulation may sometimes exist. As a result, the combinations in Eq. (2) can be classified accordingly. $C( - 1, - 1)$ and $C(1,1)$ are classified as the forward regulation relationship (denoted as $con(g_{i} ,g_{j} )$) but should fulfill the restraints in Eq. (4), $C(1, - 1)$ and $C( - 1,1)$ are classified as the reverse regulation relationship (denoted as $re(g_{i} ,g_{j} )$) but should fulfill the restraints in Eq. (5), and other combinations such as $C( - 1,0),C(1,0),C(0,0),C(0, - 1),C(0,1)$ are classified as no interaction relationships or considered noise signals.

4 $$\begin{aligned} &{\text{if }}con(g_{i} ,g_{j} ){\text{ then}} \hfill \\ &\quad \left\{ \begin{array}{l} \bullet \, ((P( - 1, - 1) + P(1,1) + P(0,0)) - (P( - 1,1) + P(1, - 1) + P(0,0))) > 0 \hfill \\ \bullet \, ((P( - 1, - 1) + P(1,1) + P(0,0)) - (P( - 1,0) + P(0, - 1) + P(1,0) + \hfill \\ \, P(0,1) + P(0,0))) > \theta \hfill \\ \end{array}\right. \hfill \\ \end{aligned}$$

5 $$\begin{aligned} &{\text{if }}re(g_{i} ,g_{j} ){\text{ then }} \hfill \\ &\quad \left\{ \begin{array}{l} \bullet \, ((P( - 1,1) + P(1, - 1) + P(0,0)) - (P( - 1, - 1) + P(1,1) + P(0,0))) > 0 \hfill \\ \bullet \, ((P( - 1,1) + P(1, - 1) + P(0,0)) - (P( - 1,0) + P(0, - 1) + P(1,0) + \hfill \\ \, P(0,1) + P(0,0))) > \theta \hfill \\ \end{array}\right. \hfill \\ \end{aligned}$$

where in Eqs. (4) and (5), $\theta$ is a threshold value between 0 and 1.

**Fig. 2:**
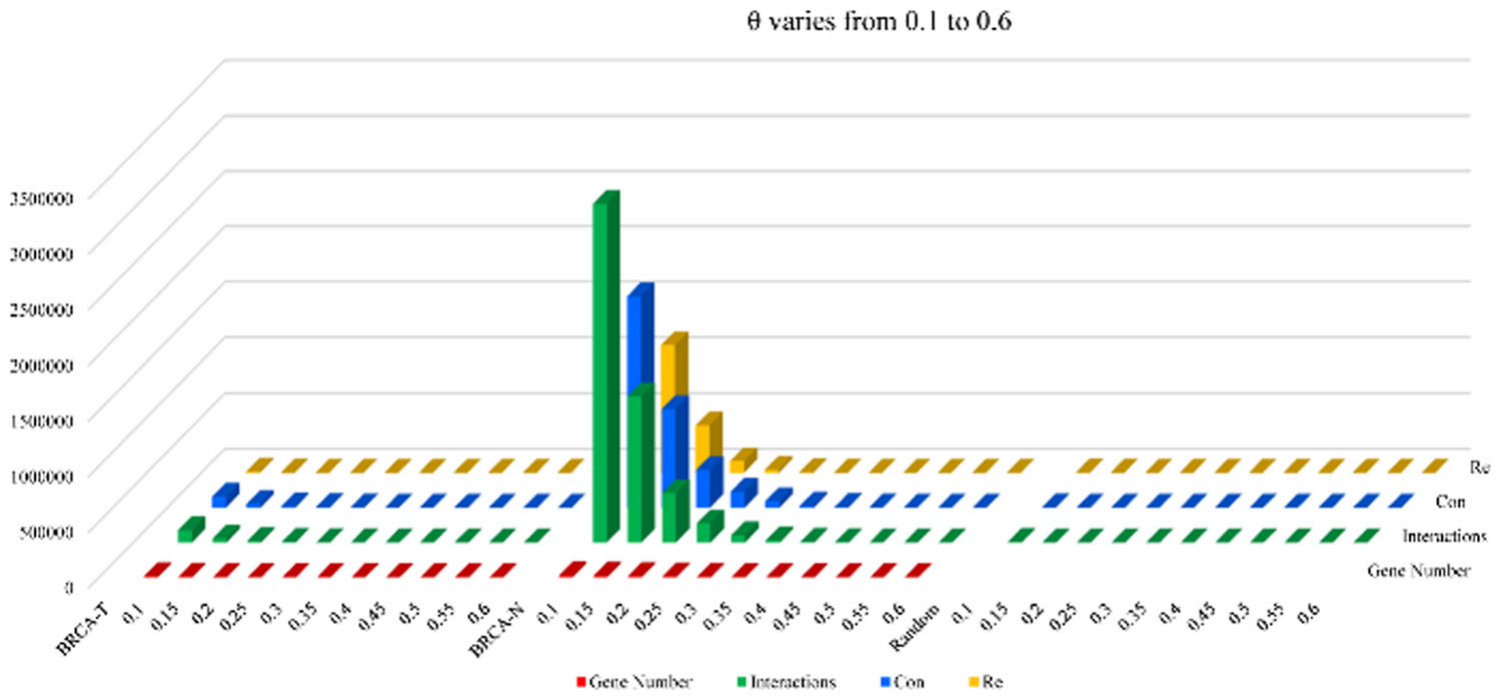
*Con* and *Re* regulations, gene number and interaction number under different $\theta$ values. BRCA-T, BRCA-N and Random represent the tumor, normal and random samples, respectively. Gene number is the number of genes contained in the filtered interactions, interactions are sum of the **Con** and **Re** interactions, **Con** signifies the interactions that are forward-regulated, and **Re** denotes interactions that are reverse-regulated

We varied $\theta$ from 0 to 0.6 (according to our calculations, no interactions exist when $\theta$ > 0.6) sin steps of 0.05, and the corresponding numbers of gene interaction pairs, genes (the number of gene of the random sample is not presented), ***Con*** regulations (forward regulation) and ***Re*** regulations (reverse regulation) detected from the tumor, normal and random samples are presented in Fig. 2. $\theta$ varies from 0.1 to 0.6. Most interactions exist between 0 and 0.1, and when $\theta$ > 0.1, almost no interactions exist in the random dataset. Therefore, we set $\theta$ = 0.1 in this study such that as many real gene interactions as possible can be filtered.

We previously hypothesized that if genes ***g***_***i***_ and ***g***_***j***_ are ***Con*** regulated then their ***Re*** regulation power under same conditions should be small, as denoted in Eqs. (4) and (5). In fact, in real organisms, forward regulations are the most common type of regulation, which is consistent with our results, as shown in Fig. 2. As shown in Fig. 2, the number of ***Con*** and ***Re*** interactions is almost equal in the random samples, which again verified the validity of our method.

**Fig. 3:**
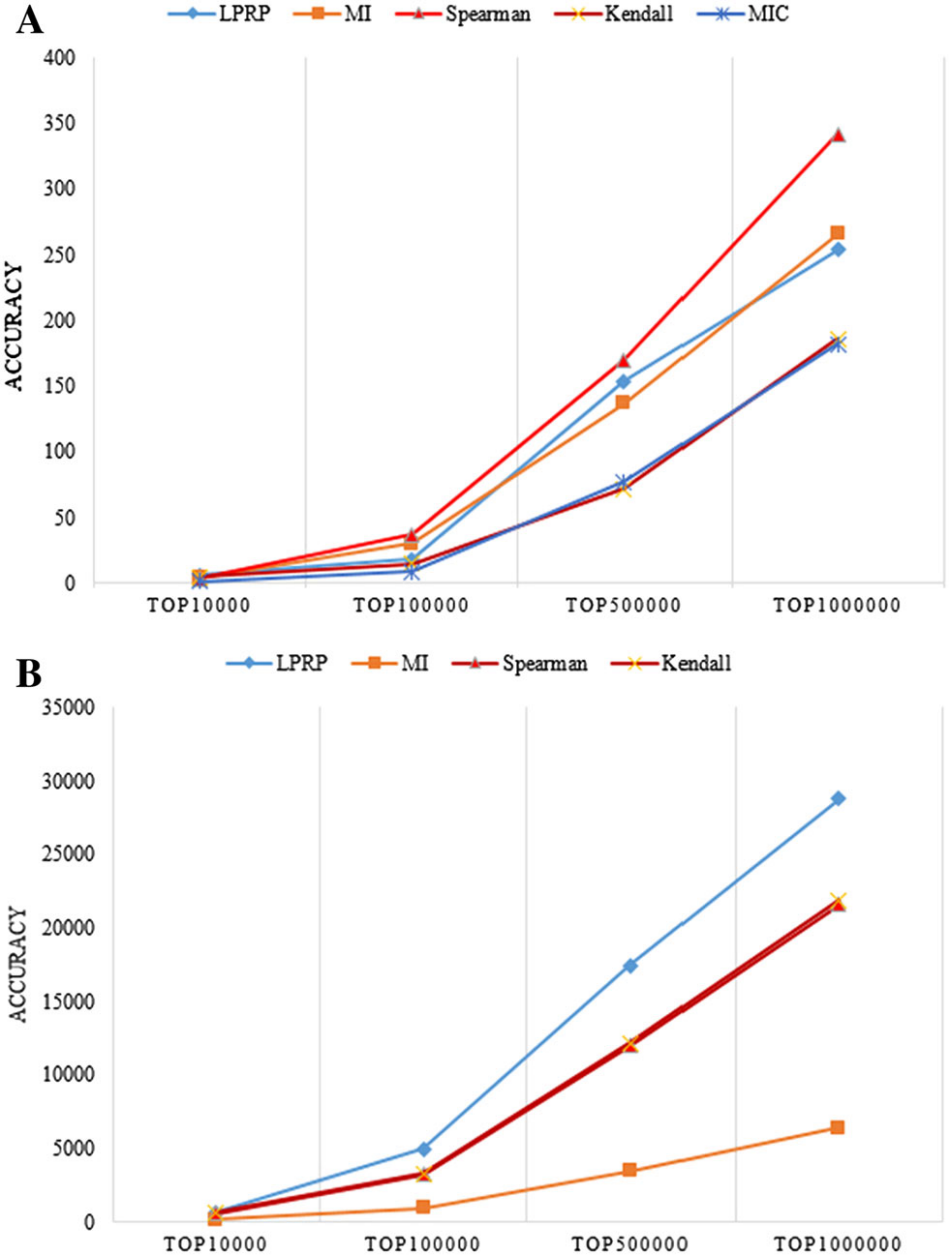
Performance comparison of the LPRP and other methods using both simulated and real datasets. ACCURACY is defined as the number of known GGIs in the top 10,000, 100,000, 500,000 and 1,000,000 interactions. The top 10,000 is the number (10,000) of GGIs filtered in each method under the given thresholds. **a** The result based on the Dream5 network 4 datasets. **b** The result based on the normal BRCA gene expression datasets. MIC is not included due to its long running time

To further validate the effectiveness of our gene interaction detection strategy, gene expression datasets from the DREAM 5 network inference challenge 4 were downloaded. After careful revision, we kept 104 samples containing 5950 genes. The golden standard network of this expression dataset was also downloaded, which contained 1994 genes and 3994 edges. The performance of the LPRP was compared with the performances of Spearman, MI, Kendall and MIC, and the results are shown in Fig. 3.

As shown in Fig. 3, the LPRP detected more known edges compared to other methods, especially in the real gene expression datasets. However, even with the LPRP of the 3994 given edges in the Dream 5 network4, only 258 edges were detected out and of the 1,896,352 edges in ***KP***, and only 28,768 edges were detected out of the top 1,000,000 filtered edges. Therefore, gene interaction pairs should not be used directly as the final edges for the network construction; instead statistical validation and a network construction strategy should be introduced to reduce the false discovery rate.

#### Statistical Validation

To validate statistical significance of the GGIs identified by the LPRP, for each gene in matrix ***D***, the order of its value (−1, 0, 1 s) across all samples was randomly shuffled. This process was repeated 1000 times, this will allow matrix ***D*** follows the same distribution as the original matrix. The LPRP was applied on all of the 1000 randomized matrices ***D***, and each time only the interactions with interaction values larger than 0.1 were filtered. First, we compared the filtered GGIs number obtained from the real datasets and the 1000 random datasets. Second, the proportion of GGIs contained in ***KP*** was calculated. Higher proportions correspond to increased LPRP effectiveness (as shown in the results section, nearly four times more GGIs contained in ***KP*** were detected by the LPRP in the real dataset compared to the random datasets). Third, because many more GGIs are filtered with $\theta$ > 0.1 in the real datasets, how large the possibility is if interactions satisfying $\theta$ > 0.1 while not annotated by ***KP*** are novel activated interactions rather than occurred by chance? To do this, the appearance times of each such interaction are counted across all GGIs generated from 1000 randomized datasets. The *p* value is defined as the proportion of appearance times to 1000 (Eq. (6)). Very few interactions had a *p* value >0.01. Smaller *p* values correspond to lower probability of the interaction occurring by chance. In this study, only GGIs with a *p* value ≤0.01 were considered.

6 $$P-{\text{value}} = \frac{{T_{\text{appear}} + 1}}{1000}$$

where $T_{\text{appear}}$ is the appearance time of one GGI in the GGIs filtered from 1000 random datasets.

#### GGI Network Construction

After statistical validation, all interactions with $\theta$ > 0.1 but *p* value >0.01 were discarded. Only interactions with $\theta$ > 0.1 and a *p* value <0.01 were used for the GGI network construction. The LPRP constructs the GGI network in three steps: raw GGI network construction, expansion and revision. The raw GGI network is constructed by using interactions that satisfy the threshold value and also contained in ***KP*** [21]. In this way, we can easily obtain a rough topology of the final GGI network without introducing much false positive gene interactions [39]. The gene interactions in ***KP*** have no direction; therefore, gene ***g***_***i***_ interacts with ***g***_***j***_ is equivalent to ***g***_***j***_ interacts with ***g***_***i***_. If either one exists in ***KP***, we selected the edge from the filtered GGIs (all the GGIs satisfy $\theta$ > 0.1 and *p* value <0.01). With these raw GGIs, the expansion and revision processes are executed alternatively until no edges remain in all of the candidate GGIs. The purpose of expansion is trying to add as much of edges left after raw construction as possible to the raw network, while revision is preventing expansion from introducing noise edges. In expansion, all of the endpoint genes of the currently not added edges are considered, and only genes that have direct interactions with genes contained in the current GGI network are attached to the current GGI network. Revision is performed only between the newly added genes after the current expansion stage. GGIs that satisfy the statistical validation but that are not attached to the current GGI network can be classified into the following three categories. (a) GGIs with both endpoint genes already contained in the current raw GGI network but not included in ***KP***. We use Eq. (7) to judge whether such GGIs should attach to the current GGI network. In Eq. (7), $Comneg(g_{i} ,g_{j} )$ represents the common neighbors of genes ***g***_***i***_ and ***g***_***j***_. If their common neighbor number is larger than the threshold value $\omega$, then we add an edge between them; otherwise, they are left unconnected. (b) GGIs with both endpoint genes not contained in the current raw GGI network. For these GGIs, because it is hard to determine whether they should be attached, we left them as undetermined in the current cycle period. (c) GGIs with only one endpoint gene included in the raw GGI network. For these GGIs, we simply attached them to the current GGI network. GGIs in (a) and (b) may correspond to novel interactions or simply interactions that occurred due to their common interacting neighbors. In revision stage, we weigh whether those GGIs should be added or discarded. As shown in Eq. (7), if their common neighbor number is bigger than the threshold value, they are attached to the current raw network. Otherwise, they are discarded.,

7 $$Comneg(g_{i} ,g_{j} ) > \omega$$

where $Comneg(g_{i} ,g_{j} )$ is the number of common neighbors of genes ***g***_***i***_ and ***g***_***j***_, and $\omega$ is the threshold value. We set $\omega$ = 1 in this study (after the raw construction stage, all the known GGIs have already been added to the network. The main purpose of the expansion and revision is to add as many edges as possible, because although these edges are not contained in the know edge interaction datasets, they all pass our statistic validation and are more likely to be real existed interactions rather than occur by chance. Hence, setting $\omega$ at a smaller value is preferable.).

## Results

**Fig. 4:**
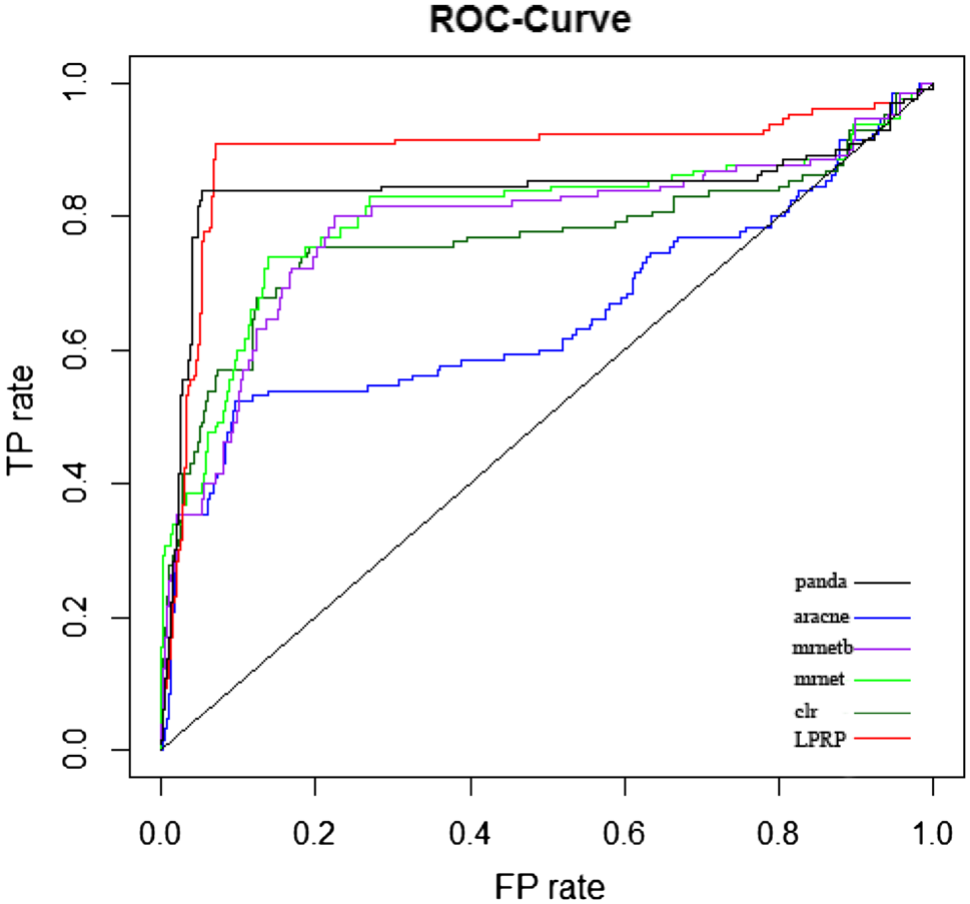
Performance comparison of the LPRP and other methods using syn.data real gene expression dataset (syn.data contained in minet R package, syn.data includes gene expression dataset and reference network). Panda [40, 41], Mrnet [42], CLR [43], ARACNE [16] and mrnetb are GGI network inference methods, all can be found in minet [44] R package

**Fig. 5:**
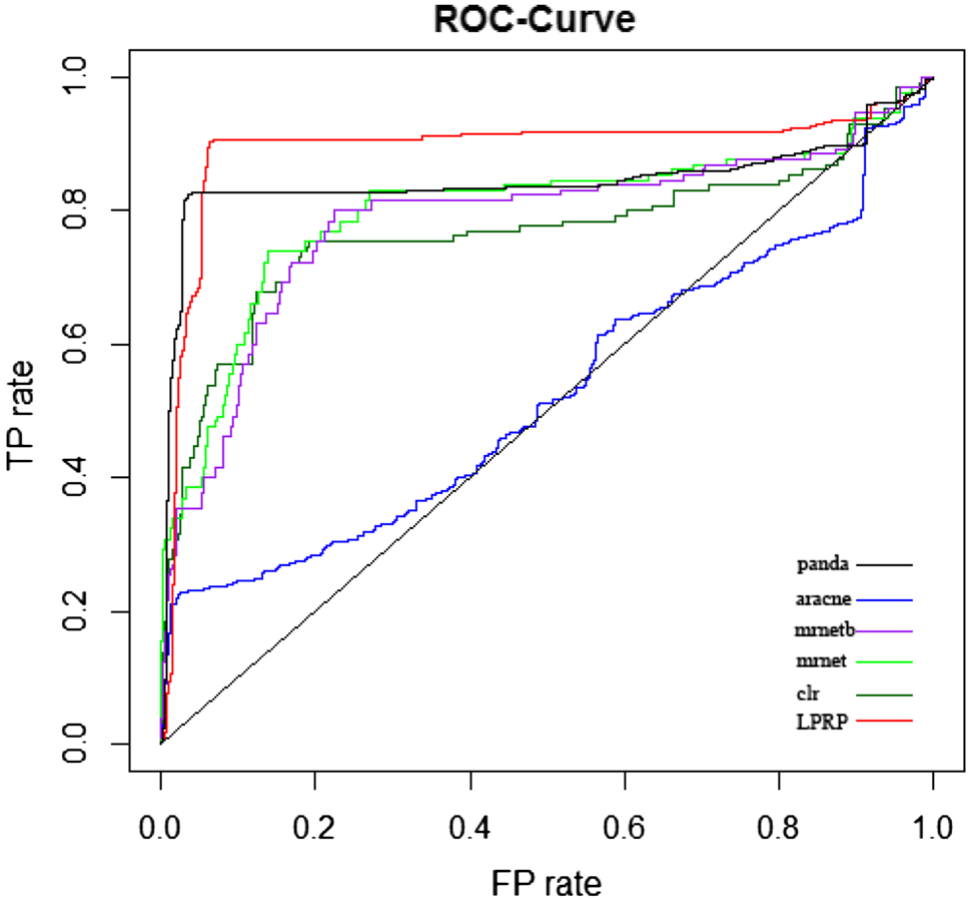
Performance comparison of the LPRP and other methods using simulated gene expression dataset (with 100 genes and 100 samples simulated by SynTren. SynTren can not only simulate gene expression but also give reference network)

Before applying LPRP on breast cancer datasets analysis, effectiveness of LPRP is evaluated using both real gene expression dataset and on simulated gene expression dataset. Results are shown in Figs. 4 and 5.

As shown in Figs. 4 and 5, LPRP performs well on both real and simulated gene expression datasets. Next, we apply LPRP on breast cancer datasets analysis and construct GGI networks of normal and tumor samples, respectively. According to our analysis, we found that the GGI network constructed from normal samples is a typical complex network. Its node degree follows the power law distribution, and its characteristic path length and network diameter are 4 and 13, respectively, these values are 8.6 and 24 for the tumor GGI network, respectively. The tumor GGI network contains fewer genes and gene interactions, and its path length is longer. In fact, its path length follows a normal distribution. Because a random mutation/alteration in cancer is more likely to inactivate rather than activate a gene, there is a large reduction in the number of genes. Recent reports have also suggested that most genetic mutations inactivate and affect tumor suppressor genes [45]. The node overlap between the two GGI networks is large (56 % of the tumor nodes are found in the normal GGI network), but only 18.4 % of the interactions present in the tumor network are found in the normal network. According to [46], cancer may be a pathway to cell survival, as in the tumor GGI network, new paths occurred and new genes were activated, and these paths and genes may play key roles in tumor initiation and development. According to James West [10], cancers are characterized globally by an increased network entropy, and the larger the network entropy corresponds to the lower system stability. Increased network diameter and a decreased clustering coefficient in the tumor network together foster such instability.

Multi-level comparisons were performed between the normal and tumor GGI networks. First, we compared the networks from the entire network perspective, including the differences in their network topology characteristics and their common and particular genes and edges. Second, clusters within the two networks were detected using MINE, and their functions were annotated using the SIGORA R [38] package and DAVID [47]. Third, the characteristics of special genes (including genes particularly expressed in tumor network, common network genes that were differentially expressed, known breast cancer genes) were compared.

### Network-Level Comparison

**Fig. 6:**
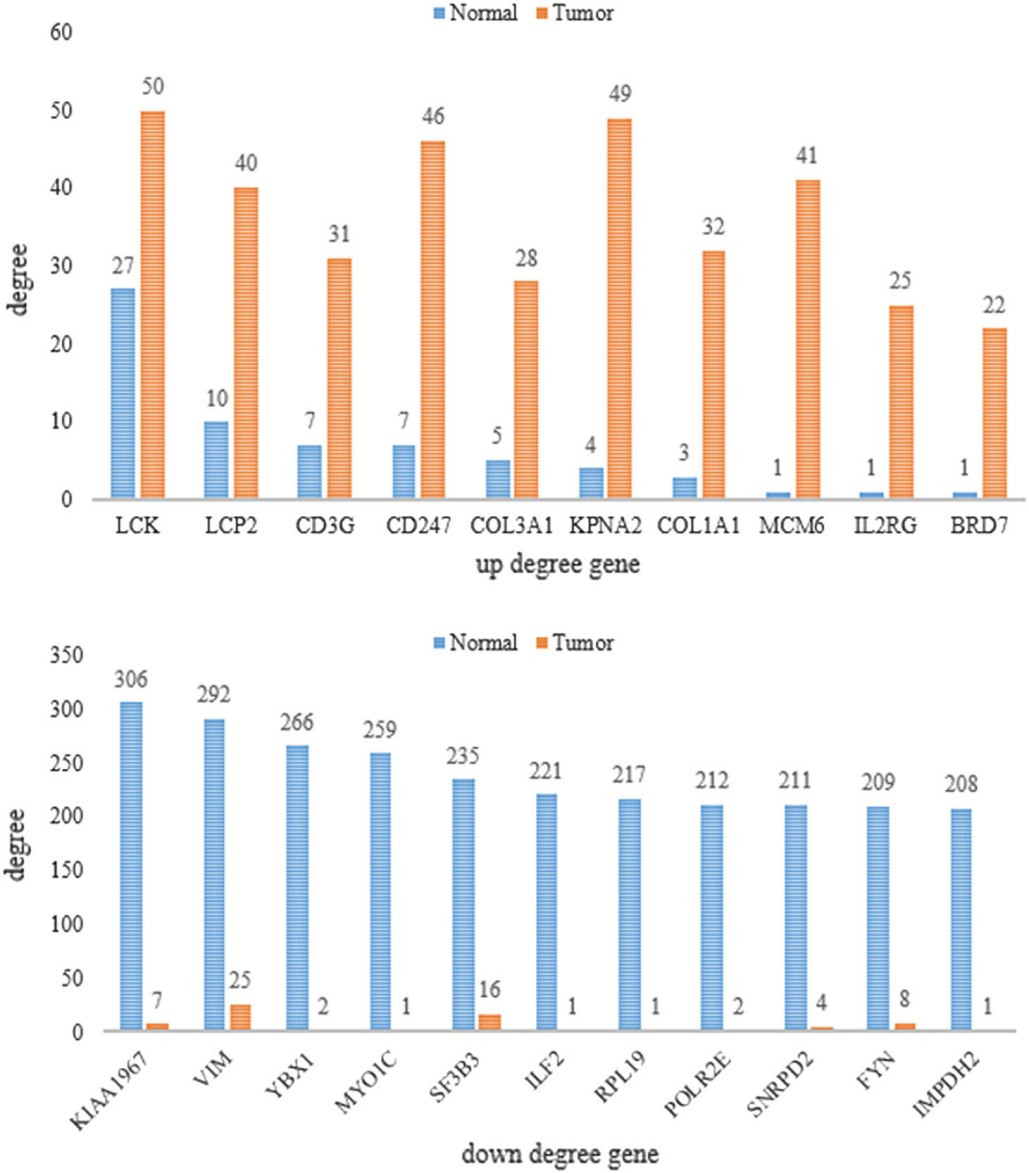
Up- and down-regulated gene node degrees in the final tumor and normal GGI networks. Normal indicates a normal GGI network, tumor indicates a tumor GGI network, up degree gene indicates the degree of genes was larger in the tumor GGI network than in the normal GGI network, and down degree gene indicates that the degree of genes was smaller in the tumor GGI network than in the normal GGI network

**Fig. 7:**
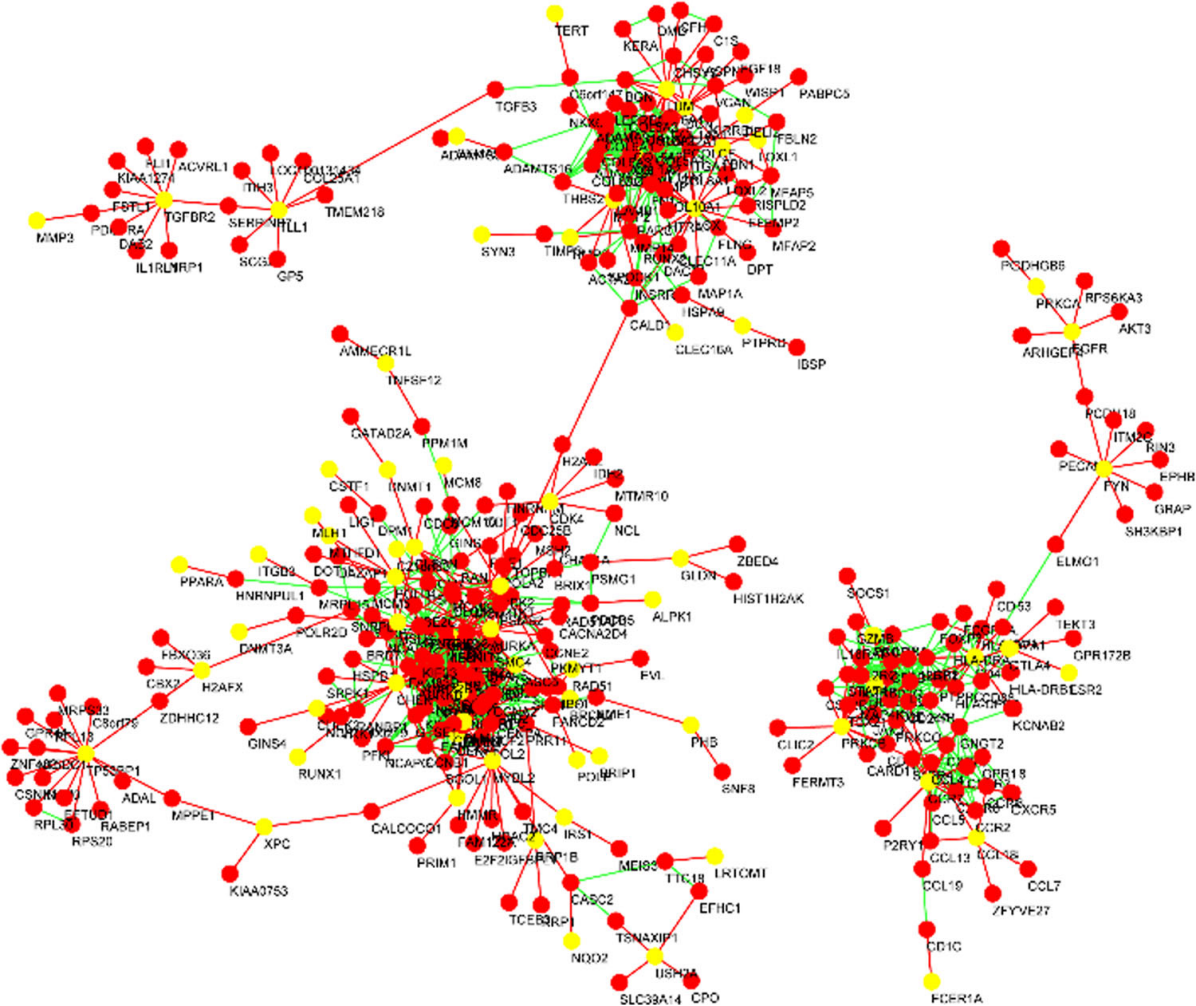
Breast disease genes and their neighbors. In this figure, known breast disease-related genes are mapped to the final tumor GGI network, and their adjacent neighbors are filtered out

By applying the LPRP on the tumor and normal BRCA datasets with $\gamma$ = 0.45 and $\theta$ = 0.1, 110,186 and 3,045,539 raw GGIs were filtered, respectively. After statistical validation using a *p* value <0.01, only 102,688 and 2,893,901 GGIs remained for the subsequent GGI network construction, which contained 10,114 and 15,714 genes, respectively. First, raw networks for both the normal and tumor GGI networks were constructed, and then the expansion and revision steps were alternately executed until no edges could be added. Detailed information is provided in Figs. 6 and 7, and the supplemental file S1. All of the network analyses were performed using Cytoscape [37, 48, 49].

### Cluster-Level Comparison

Functionally related genes rarely work in isolation; rather, they tend to form clusters and collaboratively perform complex cellular functions. By detecting clusters in both the normal and tumor final GGI networks, specific tumor functional modules can be revealed. Many cluster detection algorithms have been proposed, such as SPICi [50], GECluster [51], MCODE [33] and MINE [35]. MINE outperforms MCODE, SPICi and many other methods in identifying non-exclusive, high modularity clusters and can be easily run on Cytoscape software. MINE was run under the default parameter settings. Seventeen clusters with their node numbers greater than 5 of the tumor GGI network are listed in Table 1. The cluster functions and their enriched biological pathways were annotated using DAVID [47, 52] and the R package of SIGORA. Pathways such as the P53 signaling pathway, the cell cycle and the Jak-STAT signaling pathway are well-known cancer pathways.

**Table 1:** Functional annotation of clusters detected using MINE in the tumor GGI network

Cluster	DAVID/SIGORA annotate	Gene	Benjamini	*p* value
1	Ribosome, translational elongation	68	2.9E−125	1.1E−127
2	Cell cycle, P53 signaling pathway, DNA replication	73	9.5E−59	1.8E−61
3	Regulation of lymphocyte activation, regulation of leukocyte activation, immune response, T cell receptor signaling pathway, Jak-STAT signaling pathway	39	1.9E−21	2.3E−24
4	Eextracellular matrix, proteinaceous extracellular matrix, cell adhesion, hydroxylation, extracellular region	32	4.0E−36	4.4E−38
5	Immune response, apoptosis, regulation of apoptosis, response to virus	23	6.7E−9	3.3E−11
6	Mitotic cell cycle, chromosome, centromeric region, intracellular non-membrane-bounded organelle, chemokine signaling pathway	16	8.4E−11	4.1E−13
7	Antigen processing and presentation of peptide antigen via MHC class I	12	1.5E−13	1.5E−13
8	Antigen processing and presentation of peptide or polysaccharide antigen via MHC class II, immune response	8	1.4E−13	9.1E−16
9	IgG binding, alternative splicing	8	1.4E−8	1.5E−9
10	SH2 domain, chemokine signaling pathway	7	1.2E−4	2.4E−6
11	Protein biosynthesis, RNA transport	7	1.7E−4	1.8E−5
12		7		
13	Chemokine signaling pathway, response to wounding, Cytokine-cytokine receptor interaction	6	1.7E−8	2.2E−10
14	Epidermis development, epithelial cell differentiation, ectoderm development	5	1.9E−7	6.8E−9
15	Immune response	5	2.6E−4	6.2E−6
16	Chemokine signaling pathway, NOD-like receptor signaling pathway, sh3 domain	5	1.7E−2	5.1E−4
17	Cell cycle, DNA replication	5	2.3E−6	3.8E−7

### Gene-Level Comparison

The final tumor GGI network contained 4757 genes, 56 % (2668) of which were also contained in the final normal GGI network. The other 44 % genes may play important roles in tumor progression. The enriched KEGG pathways of the genes were analyzed using the R package of SIGORA [38]. The top 25 pathways with a *p* value <0.0008 are listed in Table 2. As shown in Table 2, many pathways are well-known tumor pathways. Because few genes and interactions are contained in the tumor GGI network, most genes in the tumor GGI network have less interacting edges compared to normal networks. Through comparative analysis node degree of those common genes, we found that most of them have same neighbor numbers in both tumor and normal GGI network; however, some genes have significant change in their degree. In gene-level comparison, we filtered such significantly changed genes and results were shown in Fig. 6.

**Table 2:** KEGG pathway enrichment analysis results

ID	Pathway	*p* value
1	Cytokine–cytokine receptor interaction	4.46E−200
2	Metabolic pathways	2.25E−34
3	Jak-STAT signaling pathway	3.24E−21
4	Protein processing in endoplasmic reticulum	1.18E−10
5	ErbB signaling pathway	8.19E−09
6	Amino sugar and nucleotide sugar metabolism	3.92E−06
7	Histidine metabolism	9.17E−06
8	Caffeine metabolism	1.05E−05
9	Glycerophospholipid metabolism	1.32E−05
10	Asthma	1.41E−05
11	Vitamin B6 metabolism	1.60E−05
12	Sulfur relay system	1.67E−05
13	Small cell lung cancer	2.35E−05
14	Cysteine and methionine metabolism	2.80E−05
15	Glycosphingolipid biosynthesis—lacto and neolacto series	3.14E−05
16	Fc gamma R-mediated phagocytosis	6.53E−05
17	Base excision repair	0.0001419
18	Synthesis and degradation of ketone bodies	0.0001611
19	Protein digestion and absorption	0.0001899
20	Porphyrin and chlorophyll metabolism	0.0002799
21	GnRH signaling pathway	0.0003071
22	Osteoclast differentiation	0.0004201
23	Alanine, aspartate and glutamate metabolism	0.0006986
24	Long-term potentiation	0.0007306
25	Glycosylphosphatidylinositol(GPI)-anchor biosynthesis	0.0007490

### Potential Breast Tumor Gene Prediction

Next, we mapped the 166 (all genes are downloaded and compiled from SNP4Disease website) known breast disease-related genes to the final tumor GGI network. These genes and their neighbor genes were filtered out, and the result is shown in Fig. 7. As shown in Fig. 7, breast genes and their neighbor genes fall into three clusters. Because genes within the same cluster tend to have similar functions, we first annotated the three clusters using DAVID, and the results are shown in Table 3. According to the functional annotation results, many of these genes contribute to cancer initiation and progression and may act as potential breast cancer genes.

**Table 3:** DAVID annotation results of the three clusters in Fig. 7

Cluster	DAVID Annotate	Gene	Benjamini	*p* value
1	Extracellular matrix, cell adhesion, blood vessel development, EGF-like region, conserved site, cell migration, pathways in cancer	77	1.0E−54	5.7E−57
2	Disulfide bond, transmembrane protein, Chemokine signaling pathway, inflammatory. Response, immune response, apoptosis	62	1.3E−14	5.9E−17
3	Cell cycle, DNA repair, regulation of cell cycle process, pathways in cancer, apoptosis, immune response	180	8.0E−41	2.7E−43

## Conclusion

In this study, both normal and tumor GGI networks were constructed under the same parameter settings, and multi-level comparisons are conducted. Results show that the tumor GGI network has larger network diameter with longer characteristic path length but a smaller clustering coefficient and much sparse network connections, which are different from those of normal GGI network. The tumor GGI network contains fewer functional modules, and many of them were enriched in known cancer-related pathways. Among the up-regulated genes, BRD7 encodes a protein that interacts with p53 and is required for p53-dependent oncogene-induced senescence, which prevents tumor growth. Among the down-regulated degree gene, KIAA1967, also known as Deleted in Breast Cancer 1 (DBC1), is a candidate tumor suppressor gene involved in breast cancer [53, 54]. Finally, by mapping known breast-related disease genes to the final tumor GGI networks, three clusters were filtered out. Because genes within the same cluster tend to have similarly functions, genes within these clusters may be potential breast cancer genes. These findings allow for a better understanding of tumor mechanisms and may have potential implications for the identification of novel drug targets.

## Electronic supplementary material

Below is the link to the electronic supplementary material.

S1 File Network characteristics of both tumor and normal GGI networks (PDF 1697 kb)
