# Supplementary material for: LPRP: A Gene–Gene Interaction Network Construction Algorithm and Its Application in Breast Cancer Data Analysis
Source: Interdiscip Sci. 2016 Sep 17;10(1):131–42. doi: 10.1007/s12539-016-0185-4 (PMC5838217; doi:10.1007/s12539-016-0185-4)

Supplementary material

Journal:
Interdisciplinary Sciences: Computational Life Sciences

Title:
LPRP: a Gene-gene interaction network construction algorithm and its application in breast cancer data analysis

Authors Information:
Lingtao Su[1,2], Xiangyu Meng (✉) [1,2], Qingshan Ma[3], Tian Bai[1,2], Guixia Liu (✉) [1,2]
1 College of Computer Science and Technology, Jilin University, Changchun, 130012, China
2 Key Laboratory of Symbolic Computation and Knowledge Engineering of Ministry of Education, Jilin University, Changchun 130012, China
3 The First Clinical Hospital of Jilin University, Changchun 130021, China
✉: liugx@jlu.edu.cn，sulingtao@163.com

Table 1 Details of the tumor and normal GGI networks.

Tumor GGI[a]	R[b]	E-1[c]	V-1[d]	E-2[e]	V-2[f]	E-3[g]	V-3[h]	F[i]
Number of nodes	1917	3779	3779	4678	4678	4757	4757	4757
Number of edges	4136	5998	6403	7349	7349	7436	7436	7436
Network diameter	21	22	22	23	23	24	24	24
Clustering coefficient	0.166	0.072	0.071	0.07	0.07	0.07	0.07	0.07
Characteristic path length	7.382	8.129	8.41	8.53	8.57	8.627	8.627	8.627
Network Figure	S25 Fig	S1 Fig		S9 Fig		S17 Fig		S26 Fig
Node degree Distribution	S25 Fig	S2 Fig		S10 Fig		S18 Fig		S26 Fig
Shortest Path Length Distribution	S25 Fig	S3 Fig		S11 Fig		S19 Fig		S26 Fig

Topological Coefficients	S25 Fig	S4 Fig		S12 Fig		S20 Fig		S26 Fig
**Normal GGI[j]**	**R**	**E-1**	**V-1**	**E-2**	**V-2**	**E-3**	**V-3**	**F**
**Number of nodes**	6280	6988	6988	7011	7011	7015	7015	7015
**Number of edges**	65009	65717	65730	65753	65753	65757	65757	65757
**Network diameter**	13	13	13	13	13	13	13	13
**Clustering coefficient**	0.255	0.223	0.223	0.223	0.223	0.223	0.223	0.223
**Characteristic path length**	3.823	4.028	0.4030	4.031	4.035	4.045	4.046	4.048
**Network Figure**	S25 Fig	S5 Fig		S13 Fig		S21 Fig		S26 Fig
**Node degree Distribution**	S25 Fig	S6 Fig		S14 Fig		S22 Fig		S26 Fig
**Shortest Path Length Distribution**	S25 Fig	S7 Fig		S15 Fig		S23 Fig		S26 Fig
**Topological Coefficients**	S25 Fig	S8 Fig		S16 Fig		S24 Fig		S26 Fig

In S25 Fig, (a) Network figure of the raw tumor GGI network. (b) Network figure of the raw normal GGI network. (c) Node degree distribution of the raw tumor GGI network. (d) Node degree distribution of the raw normal GGI network. (e) Shortest path length distribution of the raw tumor GGI network. (f) Shortest path length distribution of the raw normal GGI network. (g) Topological coefficients distribution of the raw tumor GGI network. (h) Topological coefficients distribution of the raw normal GGI network.
*In S26 Fig, (a), (d), (g), (j) are topological coefficients, shortest path length, network figure and the node degree distribution of a PPI network downloaded from DIP (http://dip.doe-mbi.ucla.edu/dip/Main.cgi) database, respectively. [a]**Tumor GGI**: network constructed with the gene interactions detected using the tumor samples. [b]**R**: raw network, [c]**E-1**: network after the first expansion, [d]**V-1**: network after the first revision, [e]**E-2**: network after the second expansion, [f]**V-2**: network after the second revision, [g]**E-3**: network after the third expansion, [h]**V-3**: network after the third revision, [i]**F**: final network. [j]**Normal GGI**: network constructed with the gene interactions detected using the normal samples.*

S1 Fig Network figure of the tumor GGI network after the first expansion.

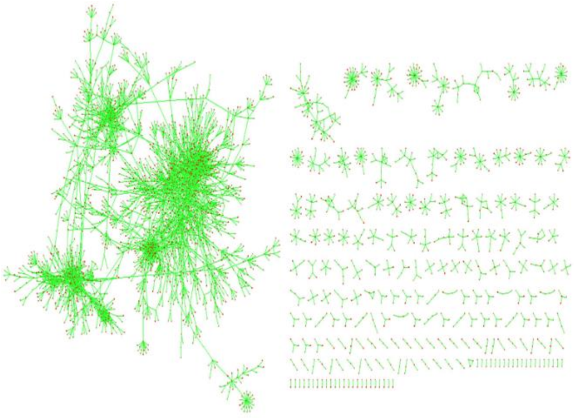

S2 Fig Node degree distribution of the tumor GGI network after the first expansion.

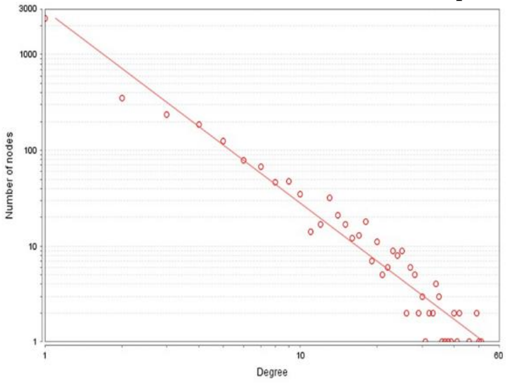

S3 Fig. Shortest path length distribution of the tumor GGI network after the first expansion.

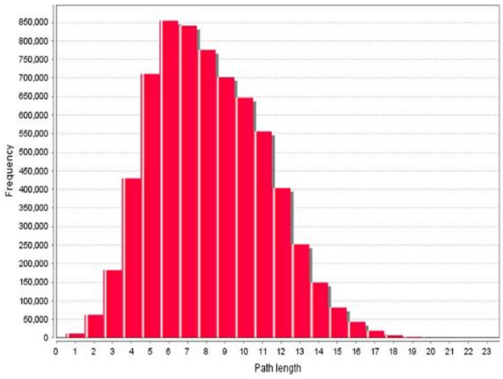

S4 Fig. Topological coefficients distribution of the tumor GGI network after the first expansion.

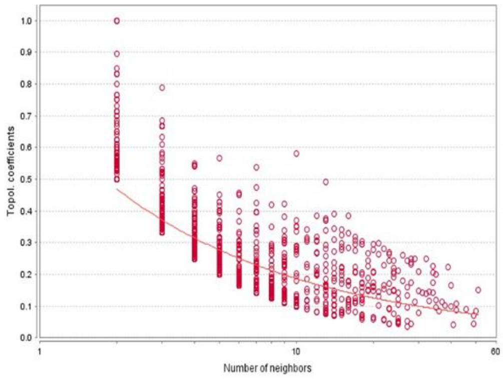

S5 Fig. Network figure of the normal GGI network after the first expansion.

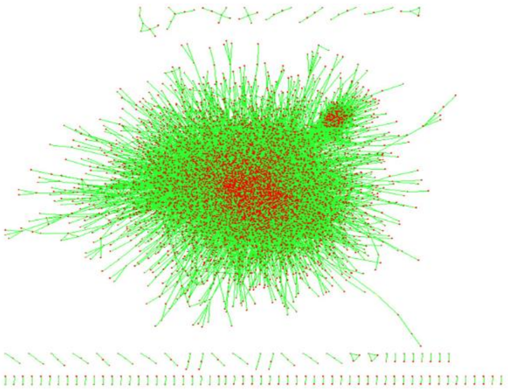

S6 Fig. Node degree distribution of the normal GGI network after the first expansion.

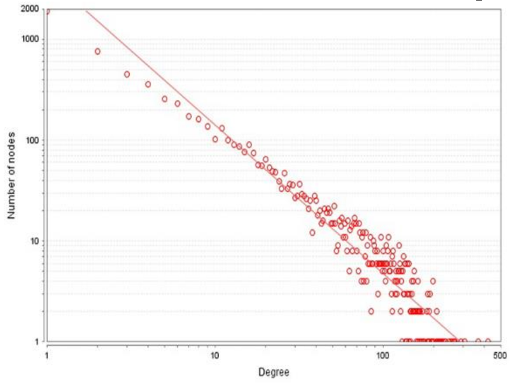

S7 Fig. Shortest path length distribution of the normal GGI network after the first expansion.

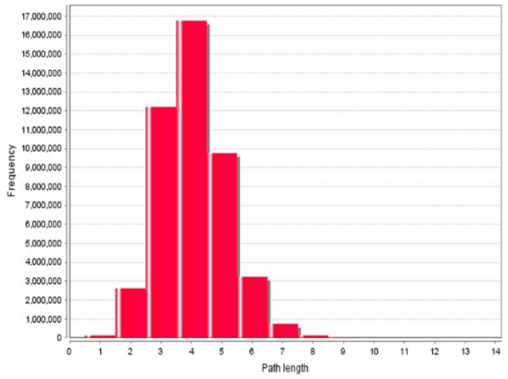

S8 Fig. Topological coefficients distribution of the normal GGI network after the first expansion.

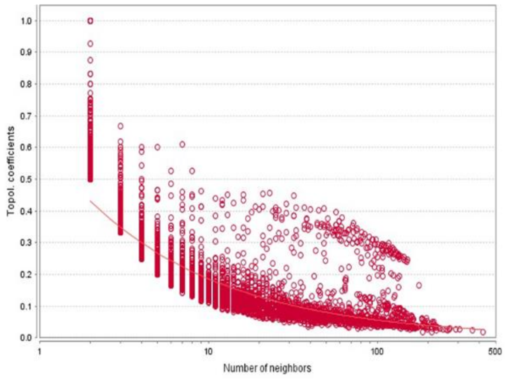

S9 Fig. Network figure of the tumor GGI network after the second expansion.

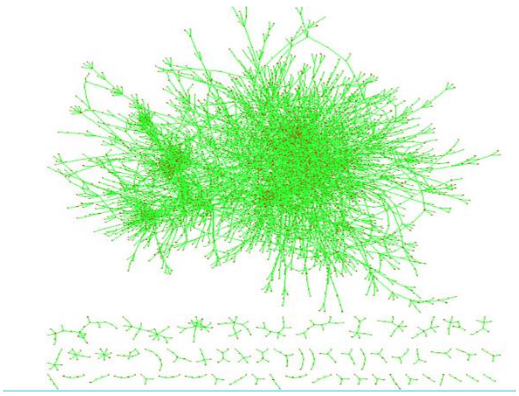

S10 Fig. Node degree distribution of the tumor GGI network after the second expansion.

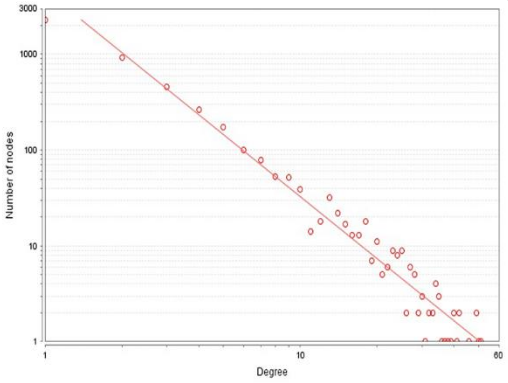

S11 Fig. Shortest path length distribution of the tumor GGI network after the second expansion.

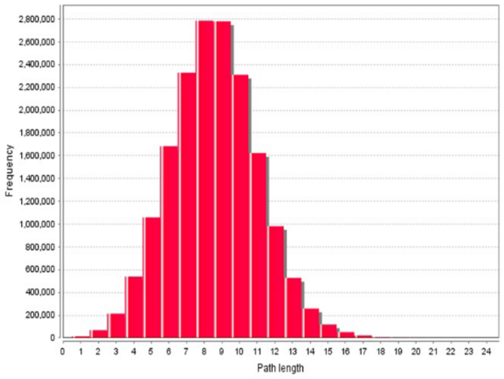

S12 Fig. Topological coefficients distribution of the tumor GGI network after the second expansion.

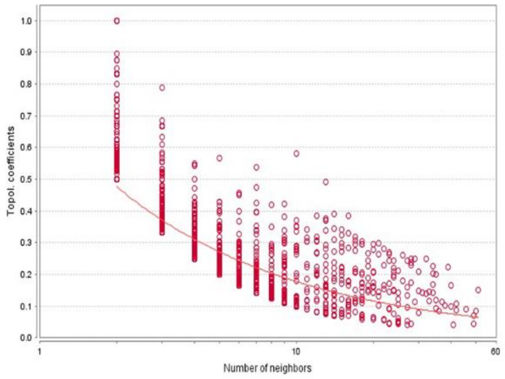

S13 Fig. Network figure of the normal GGI network after the second expansion.

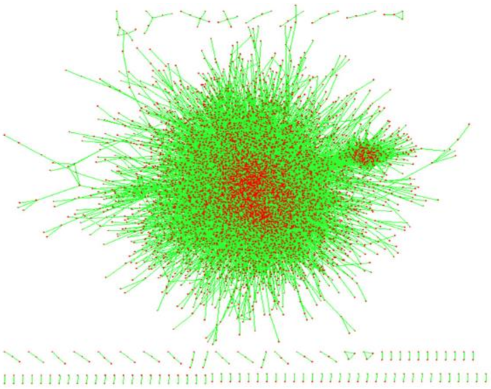

S14 Fig. Node degree distribution of the normal GGI network after the second expansion.

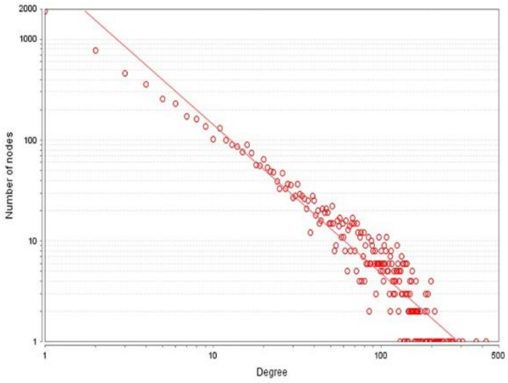

S15 Fig. Shortest path length distribution of the normal GGI network after second expansion.

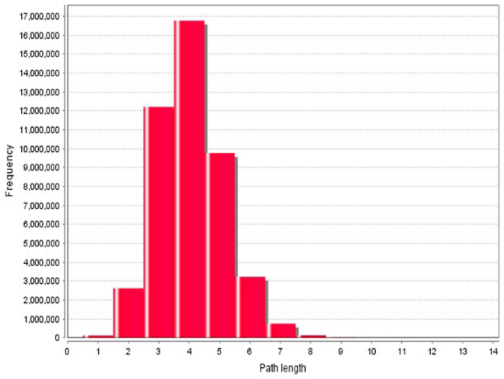

S16 Fig. Topological coefficients distribution of the normal GGI network after the second expansion.

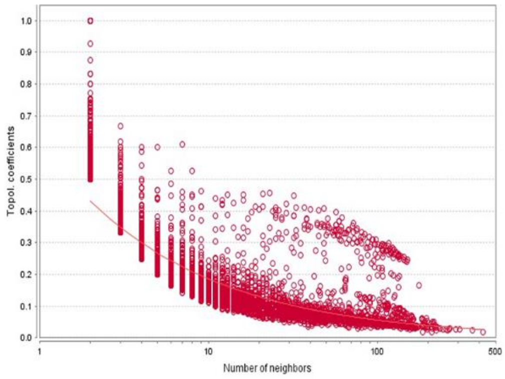

S17 Fig. Network figure of the tumor GGI network after the third expansion.

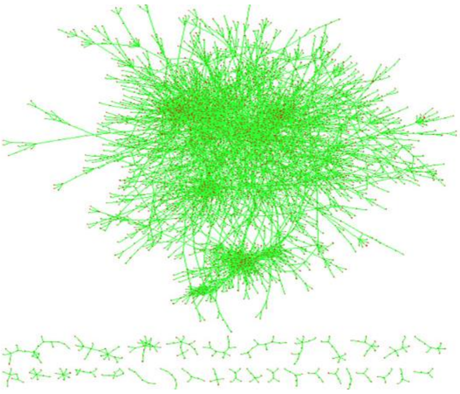

S18 Fig. Node degree distribution of the tumor GGI network after the third expansion.

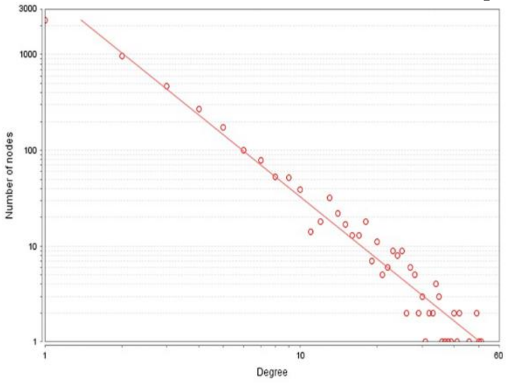

S19 Fig. Shortest path length distribution of the tumor GGI network after the third expansion.

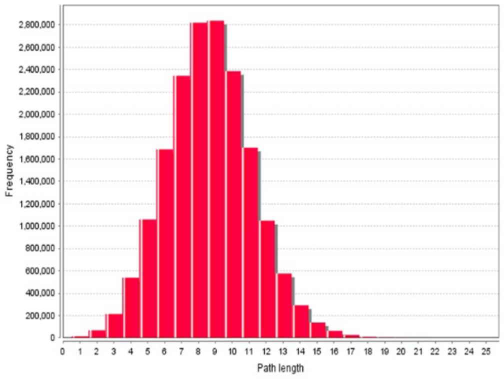

S20 Fig. Topological coefficients distribution of the tumor GGI network after the third expansion.

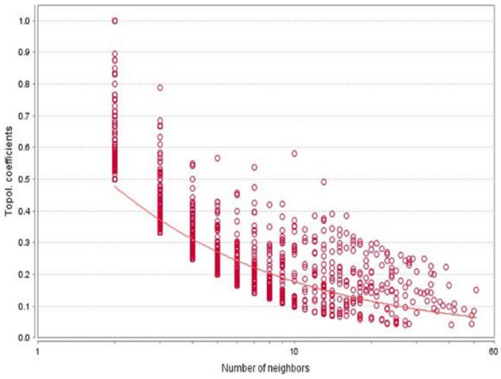

S21 Fig. Network figure of the normal GGI network after the third expansion.

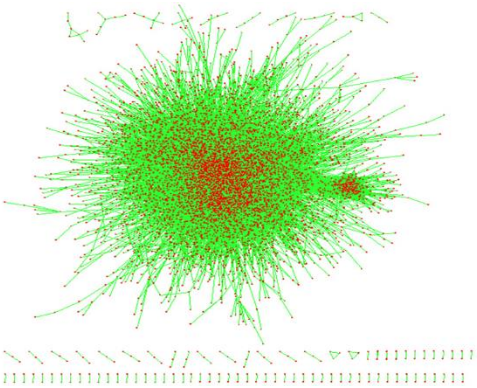

S22 Fig. Node degree distribution of the normal GGI network after the third expansion.

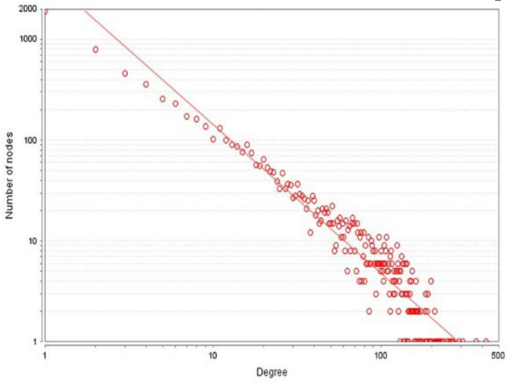

S23 Fig. Shortest path length distribution of the normal GGI network after the third expansion.

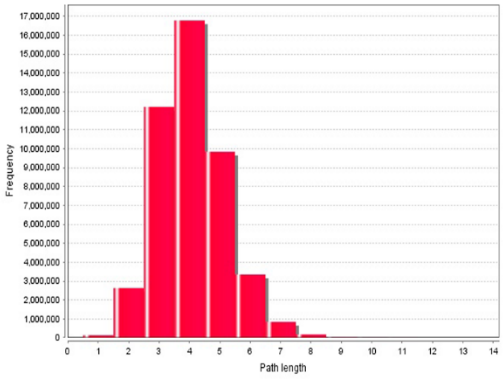

S24 Fig. Topological coefficients distribution of the normal GGI network after the third expansion.

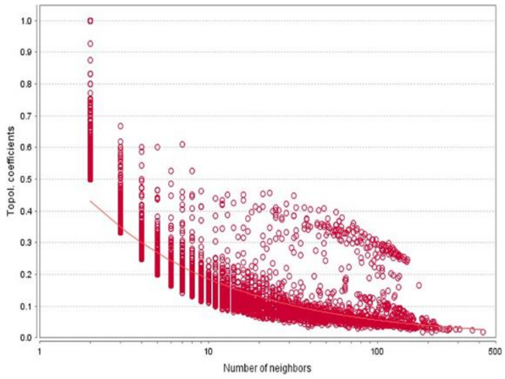

S24 Fig. Characteristic comparison of the tumor and normal raw GGI networks.

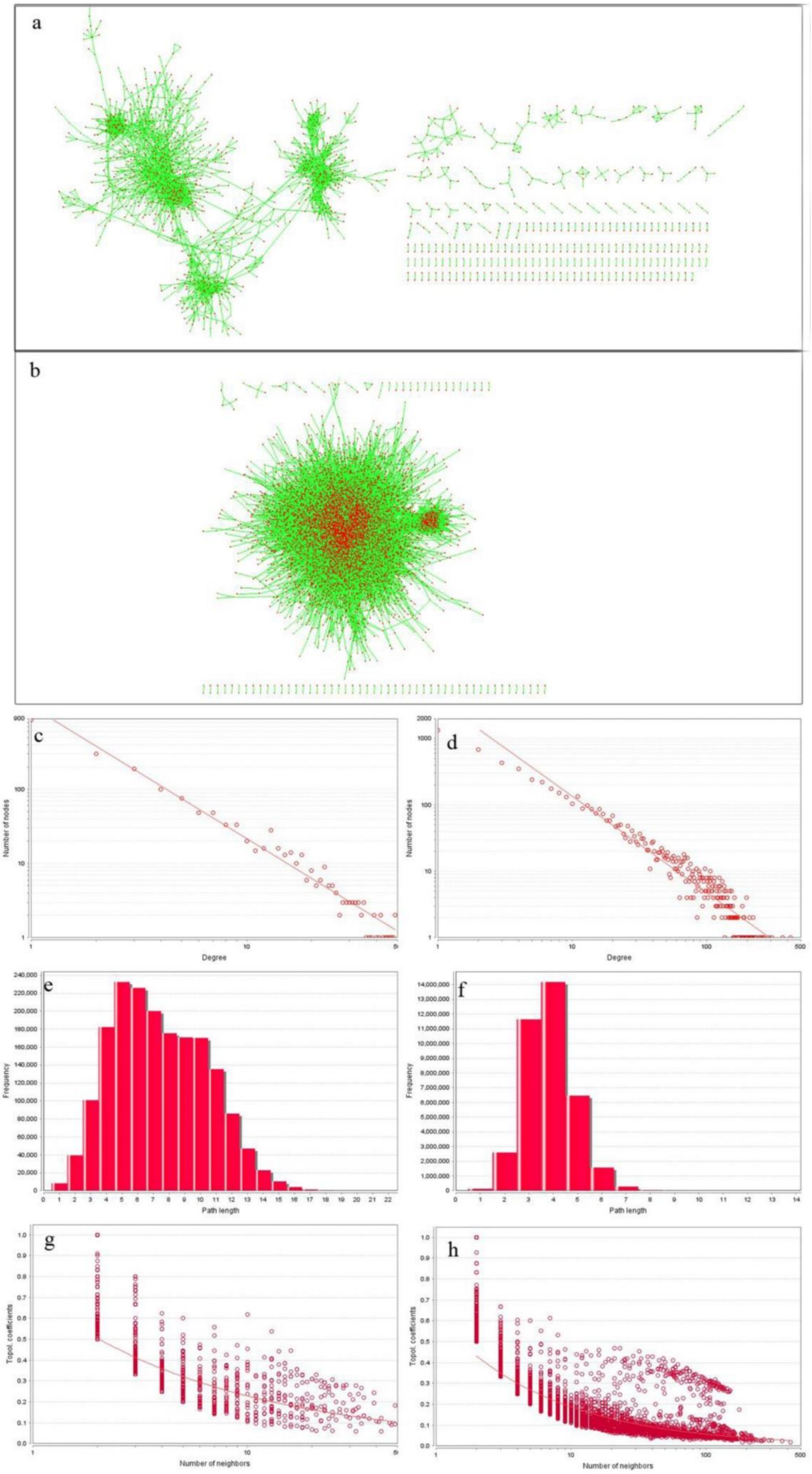
a
b
c
d
e
f
g
h
Number of nodes
Degree
Frequency
Path length
Topol. coefficients
Number of neighbors

S26 Fig. Characteristic comparison of the tumor, normal, and final PPI networks.

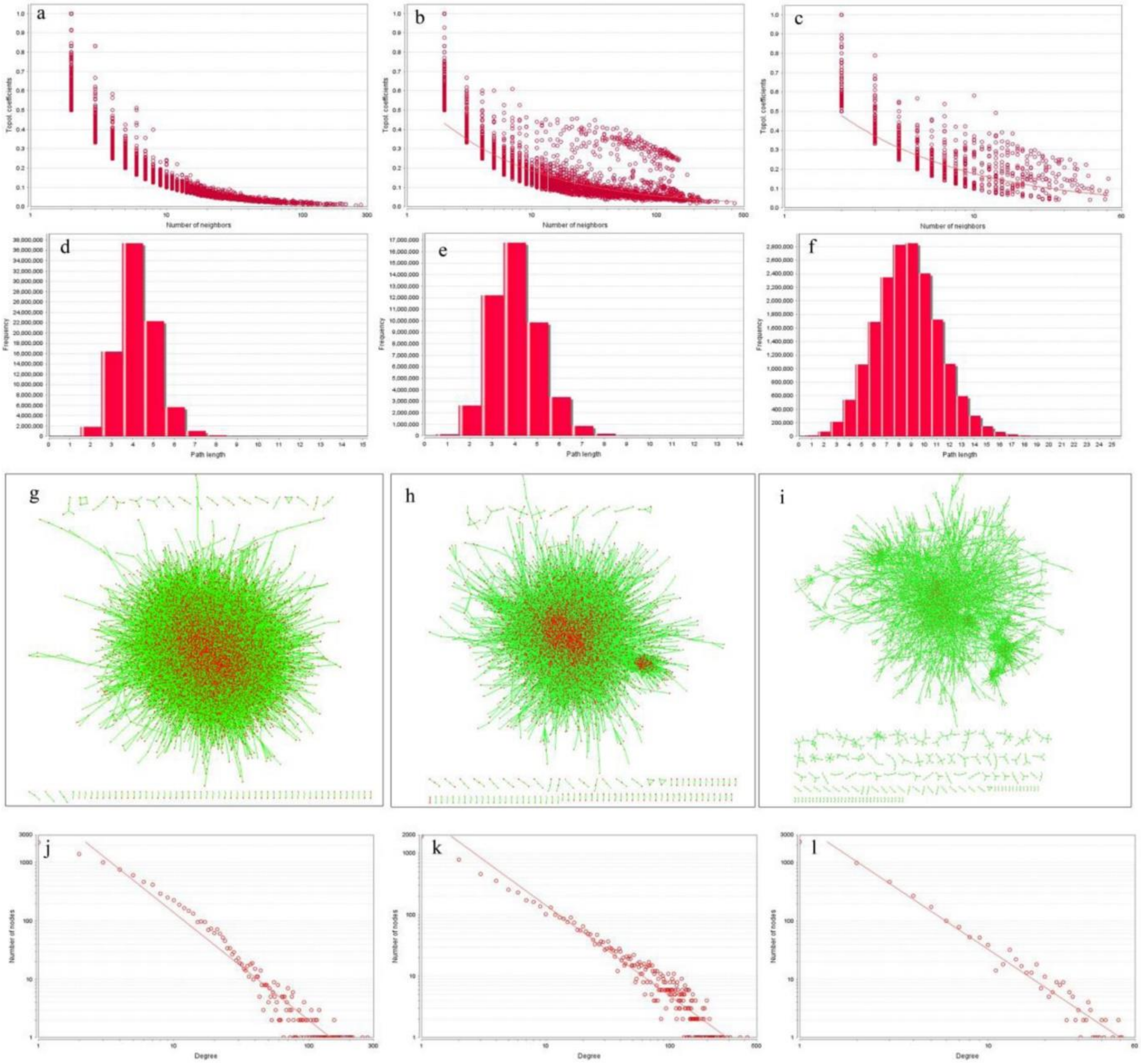

Supplement: Supplementary file 1 — Network characteristics of both tumor and normal GGI networks (PDF 1697 kb) [file 12539_2016_185_MOESM1_ESM.pdf]
